# The Quantum Mechanics of a Rolling Molecular “Nanocar”

**DOI:** 10.1038/s41598-018-33023-8

**Published:** 2018-10-05

**Authors:** Oscar E. Fernandez, Mala L. Radhakrishnan

**Affiliations:** 1 Wellesley College, Department of Mathematics, Wellesley, MA 02482 USA; 2 Wellesley College, Department of Chemistry, Wellesley, MA 02482 USA

## Abstract

We formulate a mathematical model of a rolling “molecular wheelbarrow”—a two-wheeled nanoscale molecular machine—informed by experiments on molecular machines recently synthesized in labs. The model is a nonholonomic system (briefly, a system with non-integrable velocity constraints), for which no general quantization procedure exists. Nonetheless, we successfully embed the system in a Hamiltonian one and then quantize the result using geometric quantization and other tools; we extract from the result the quantum mechanics of the molecular wheelbarrow, and derive explicit formulae for the quantized energy spectrum. We also study a few variants of our model, some of which ignore the model’s nonholonomic constraints. We show that these variants have different quantum energy spectra, indicating that in such systems one should not ignore the nonholonomic constraints, since they alter in a non-trivial way the energy spectrum of the molecule.

## Introduction

The awarding of the 2016 Nobel Prize in Chemistry for the “design and synthesis of molecular machines” is evidence of the immense interest in—and importance of—molecular machines (single molecules consisting of functional components). Indeed, research on the manipulation and control of molecular machines has advanced significantly over the past two decades. In particular, experiments have demonstrated how some “nanomachines” deposited on (typically metallic) surfaces can be moved by applying a voltage via a scanning tunneling microscope (STM) tip (see^1^ for a recent review). In most of these examples, molecules hop from one adsorption site to the next^2^. But some studies document more intriguing motion: *rolling*.

One of the earliest examples of a rolling nanomachine (we will refer to such molecules as “nanovehicles”) is that of a carbon nanotube rolled on a graphite surface using an AFM tip^3^. More recently, a variety of nanovehicles featuring wheels and axles—*nanocars*, if you will—have been synthesized and shown to exhibit rolling. In^4^, for example, a single wheel-dimer molecule (C_44_H_24_) was manipulated with an STM tip to induce rolling motion. In^5^ researchers reported “rolling a double-wheel molecule on an Au(111) surface over a short path”. And in another example^6^, a four-“paddle-wheeled” nanocar was synthesized and reported to roll in a fairly linear manner upon a metallic surface; each 360-degree wheel rotation was achieved via a series of alternating electronic and vibrational excitations.

These examples of rolling nanovehicles are intriguing because while the mechanics of rolling are well-understood at the macroscopic scale—this is the subject of *nonholonomic mechanics*—there is no known theory of rolling at the nanoscale (i.e., no “quantum nonholonomic mechanics”). Recent progress was made in^7^, where geometric quantization and other results were used to propose a quantization scheme for certain nonholonomic systems called *conditionally variational*. Conditionally variational nonholonomic systems have an associated Hamiltonian system whose Hamiltonian mechanics reproduce the nonholonomic dynamics when the initial conditions satisfy the nonholonomic constraints^7^. This makes it possible to quantize this associated Hamiltonian system and extract the quantum mechanics of the original nonholonomic system from the result.

In this paper we formulate—and quantize—a mathematical model of the “molecular wheelbarrow” studied in^5^, a nanocar consisting of two wheels (discussed in more detail in the Methods section). As we show, the nonholonomic system obtained is conditionally variational. We then follow the approach taken in^7^—suitably modified to address new challenges encountered—and use geometric quantization, along with prior results, to quantize our molecular wheelbarrow model. The results yield detailed information on the quantum mechanics of the system, as well as quantifiable differences in the quantum mechanics of the rolling vs. non-rolling versions of the molecular wheelbarrow. This latter point has important ramifications for anyone working with nanomachines and measuring their spectroscopic properties, since it suggests that one should not ignore a rolling nanovehicle’s nonholonomic constraints in the course of analyzing the quantum mechanics of the system.

## Results

### On the construction of the model

**Figure 1:**
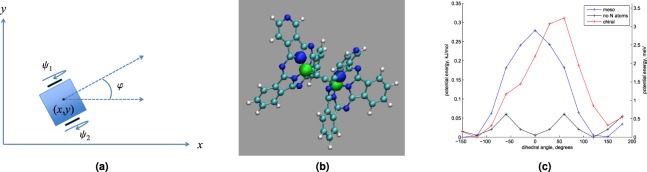
(**a**) Classical model of a two-wheeled carriage. (**b**) Model of the meso isomer of the double-wheeled molecule from^5^. Green, blue, cyan, and white atoms are boron, nitrogen, carbon, and hydrogen atoms respectively. The large spheres are the four atoms used to define the dihedral angle ${\theta }_{{\rm{d}}}={\psi }_{1}-{\psi }_{2}$ used to measure internal rotation. (**c**) Relative potential energy as a function of intramolecular rotation, as measured by *θ*_d_. For each curve, the minimum energy is set to zero. Curves are shown for the meso and chiral isomers as well as a model in which N tags have been replaced by CH groups.

We began with a well-known model of a two-wheeled carriage^8^ rolling without slipping on the *xy*-plane (Fig. 1(a)). We label the rotation angles of the two wheels by *ψ*_1_ and *ψ*_2_, and the distance between the two wheels by 2*w*. (We assume that the center of mass of the ensemble is midway on the line connecting the center of the two wheels.) We denote the position of the center of mass by (*x*, *y*), the total mass of the system by *m* (we assume both wheels have equal mass), the moment of inertia of the whole system about a vertical line through the *xy*–plane by *I*_1_, the common radius of the wheels by *a*, and the axial moment of inertia of each wheel by *I*_*w*_.

Next, we chose the boron-subphthalocyanine double-wheel molecule synthesized in^9^ and manipulated in^5^ by the tip of an STM on a gold surface to develop our mathematical model of the molecular wheelbarrow. Although the molecular motion achieved in^5^ was mainly pushing and sliding, as mentioned in the Introduction, the authors reported “rolling a double-wheel molecule on an Au(111) surface over a short path.” (The authors also report experimental evidence that suggested relative rotation of the wheels upon the surface.) The molecule, shown in Fig. 1(b), consists of two boron-subphtalocyanine moieties (the wheels) connected by a linear carbon axle. (Its symmetry suggests it roughly satisfies the center of mass assumption of the two-wheeled carriage model). Each moiety contains a nitrogen atom placed on one of the “blades” to serve as a tag for monitoring relative internal rotation. STM manipulation of this molecular wheelbarrow causes excitation resulting in higher energy states that resolve to local minima upon rolling.

To generate a coarse model of the potential energy curve, *ab initio* calculations were performed on the molecule using the Gaussview/Gaussian (Gaussian, Inc., Wallingford, CT) and the ORCA^10^ software packages. The true potential energy of the experimental system would include both changes in intramolecular interactions upon relative wheel rotation and interactions with the surface, but we will ignore the latter class of interactions to demonstrate more clearly, in a controlled manner, the energetic consequences due *only* to the *geometric* constraints of rolling on a flat surface. Including explicit surface interactions would confound these effects when compared to the vehicle rolling in free space (considered in the Special Cases of Interest subsection of the Methods section). The resulting potential energy curves (with the zero of energy set to the minimum of each curve) are shown in Fig. 1(c). (See the Methods section for more details.) We show in the Methods section that this potential is well-modeled by $V(\phi )=\alpha [1+\,\cos (2w\phi /a)]$, where *α* is the maximum value of the potential curve in Fig. 1(c) and *φ* is defined in Fig. 1(a). Adding this potential to the model for the two-wheeled carriage yielded our working model for the molecular wheelbarrow—the nonholonomic system (5). (Section A of the Supplemental Appendix reviews nonholonomic systems).

### On the quantization of the model and implications for the molecular wheelbarrow described in Nickel *et al*.

In the Methods section we detail the quantization of the model. The main results are the following^5^.

- The nonholonomic system constituting the molecular wheelbarrow model, (5), is shown to be conditionally variational and an associated Hamiltonian system is derived (c.f., (8)) whose Hamiltonian mechanics reproduce the nonholonomic dynamics when the initial conditions satisfy the nonholonomic constraints.
- The associated Hamiltonian system is shown to be quantizable via geometric quantization. Notably, the quantum Hamiltonian operator acquires a constant term proportional to the Ricci scalar curvature of the Riemannian metric associated with the Hamiltonian (8) (c.f., (15)), and the nonholonomic constraints associated with the classical wheelbarrow model are imposed at the quantum level only on average (c.f., (18)).
- Explicit formulae for the wave functions (21, 23, 29) and the energy spectra (26, 28) of the quantization of (5) are derived. Notably, the ground state energy (28) is positive in the nonzero potential case (other cases are described below) and contains the aforementioned curvature correction term, and rolling energies are quantized (c.f., (26)).
- The presence (or not) of rolling constraints and/or potential clearly alters the quantum mechanics of the system. Specifically, in the Methods section we compare the quantizations of five variations of our model. These are the wheelbarrow: without rolling constraints (we call this the *no rolling case*), with nonholonomic rolling constraints (we call this the *rolling, nonholonomic case*), and with holonomic rolling constraints (we call this the *rolling, holonomic case*). (We also consider zero- and nonzero potential variants for the first two cases.) As we discuss in the Methods section, in the rolling, nonholonomic case the wheelbarrow rolls along a circular trajectory; in the rolling, holonomic case it rolls along a linear trajectory.
- We applied our results to estimate the ground state energy of the molecular wheelbarrow described in^5^. Table 1 summarizes the ground state energies of the aforementioned five special cases.

**Table 1:** Expressions for the ground state energies of five special cases of the molecular wheelbarrow model; numbers in parentheses are corresponding predicted energies (in eV) of the molecular wheelbarrow studied in^5^.

	No Rolling	Rolling, Nonholonomic	Rolling, Holonomic
Zero Potential:	${\tilde{E}}_{\mathrm{(0,0,0)}}^{\mathrm{nr},0}=0$	${\tilde{E}}_{\mathrm{(0,0)}}^{\mathrm{nh},0}=-\,\frac{m{a}^{2}{\hslash }^{2}}{24\beta J}$ (−6 × 10^−9^)	${\tilde{E}}_{0}^{h,0}=0$
Nonzero Potential:	${\tilde{E}}_{\mathrm{(0,0,0)}}^{{\rm{nr}}}\approx \hslash \sqrt{\frac{\alpha }{I}}$ (3 × 10^−5^)	${\mathop{E}\limits^{ \sim }}_{(0,0)}\approx \frac{w\hslash }{a}\sqrt{\frac{\alpha }{\beta }}-\frac{m{a}^{2}{\hslash }^{2}}{24\beta J}$ (1 × 10^−5^)

## Discussion

The paper’s main contribution is the quantization of a nonholonomic system that can qualitatively model nanocars reported in the research literature (specifically, that of^5^). As mentioned in the Introduction, there is no known theory of quantum nonholonomic mechanics. Despite this, we were able to embed our mathematical model of the system into a larger Hamiltonian system—by leveraging the conditionally variational nature of the classical system—and then employ geometric quantization to quantize the resulting Hamiltonian system. We then extracted the quantum mechanics of the original, nonholonomic system by imposing the nonholonomic constraints at the quantum level. In our formalism, this amounts to imposing the constraints at the quantum level only on average. This is the approach favored in^11^ and references therein, since it respects the expected fluctuations in momenta of the quantum system. This feature of our approach is also reflected in the experimental evidence showing that nanocars do not always “roll without slipping”^6^, meaning that at the quantum level the nonholonomic constraints may not always be satisfied throughout the entire motion.

Once we quantized our model, several noteworthy results emerged. In particular, we found that the ground state energy (in the nonholonomic cases) acquired the constant term −$m{a}^{2}{\hslash }^{2}\mathrm{/(24}\beta J)$. This resulted from the additional curvature term in (15). That this correction leads to a negative ground state energy in the zero potential nonholonomic case (c.f., Table 1) agrees with our model of the molecular wheelbarrow as a bound system—a nonholonomic system moving on a flat surface. (We will shortly further discuss this negative ground state energy.) We also derived surprising results by considering the aforementioned four variants of the wheelbarrow system. We obtained different expressions for the energy spectra and ground state energies (c.f., Table 1), revealing four new insights. Firstly, the extra term in the quantum energy due to the curvature correction is present only in the nonholonomic models of the wheelbarrow (c.f. (26) and (32)). Secondly, the quantized energies of the rolling models are not the same as the quantized energies of the no rolling models; for instance, in addition to differing in the presence of the curvature correction, (26) and (30) differ in that *β* has been replaced by *I*. This, along with the facts that *β* > *I* (since *β* = *I* + *ma*^2^) and that *β* appears in the denominator of the ground state energy equations (c.f., Table 1) implies that, holding the potential fixed, the ground state energies in the rolling, nonholonomic case are *lower* than the ground state energies in the no rolling case. (This is easily seen from Table 1). Thirdly, even in the zero potential case the quantum energies of the rolling vs. no rolling models are not the same (c.f. (31–33)). Fourth, the presence of the nonholonomic constraints also changes the energy spacings. This is most easily seen by comparing (31) and (32). Holding *j* constant, allowing only *k* or *l* to vary produces different energy spacings because the latter equation’s denominator in the *l*^2^ fraction is *β* and the former’s is *I*. Different energy spacings imply different spectroscopic behavior between chemically identical rolling and non-rolling molecules, a prediction that future experimentation could potentially explore.

These results have important ramifications for the quantum mechanics of nanocars. Let us highlight two in particular. Firstly, when we applied our results to the molecular wheelbarrow, even in the zero potential case there was a difference in the ground state energies and energy spacings of the constrained vs. unconstrained systems (c.f., Table 1) stemming ultimately from the presence of nonholonomic constraints inherent in the original wheelbarrow model (2). This suggests that one should take care in analyzing the quantum mechanics of nanovehicles that are known (experimentally) to “roll,” since ignoring the presence of the constraints (holonomomic or nonholonomic, which mathematically encode the rolling) may lead to a different energy spectrum. Secondly, the transition from the nonzero- to the zero-potential nonholonomic case contains interesting insight. The periodicity of the potential may be predicted by the symmetry group of the wheels, whereas the amplitude (*α*) is determined by the extent to which the wheels interact with each other, which depends both on axle length and the electrostatic properties of the wheels. In our case, the more symmetric the molecule—and in particular its wheels—the lower the *α*-value (as reflected in Fig. 1(c), see Methods). One can imagine approaching the limit of a molecule whose intramolecular rotation generates no potential by using apolar wheels with near cylindrical symmetry about the axle (perhaps “buckyball” or other carbon-derived moieties may approach this limit). This *α* = 0 molecule would feature the negative ground state energy ${\mathop{E}\limits^{ \sim }}_{(0,0)}^{\text{nh},0}$ from (32) in the nonholonomic case. Therefore, one can understand that negative energy as the theoretical limit of the ground state energies in the nonholonomic case as the molecule becomes more symmetric. These observations suggest that one might be able to use the model systems in this work to qualitatively predict or design energy spectrum features as a function of molecular properties.

Finally, let us note that our results are based on the quantization of our (nonholonomic) model of the molecular wheelbarrow via the quantization of its (classical) associated Hamiltonian system. A simpler approach would have been to quantize the Hamiltonian system defined by the second-order *φ* and *θ* system in (3) and then require the resulting wave functions to satisfy the nonholonomic constraints at the quantum level (enforced via (16)). This produces results identical to ours except for one difference: the *R*-correction term in (17) is zero. (The Appendix contains the calculations.) Physically, this has the effect of shifting up the quantized energies in the nonholonomic case by $m{a}^{2}{\hslash }^{2}\mathrm{/(24}\beta J)$; in particular, it results in a ground state energy in the zero-potential nonholonomic case of ${\tilde{E}}_{(0,0)}^{0}=0$, compared to the negative energy we found (c.f., Table 1). Lacking a full theory of quantum nonholonomic mechanics, determining which set of predictions is correct could become a potentially challenging and interesting experimental matter—synthesizing highly symmetric molecules or wheels (yielding *α*-values close to zero) and determining the feasibility of indirectly measuring relative ground state energies through energy transitions would be the next steps toward settling the matter. We chose our approach because it is grounded on the body of literature on geometric quantization and associated Hamiltonian systems, while the simpler approach above has no overarching theoretical foundation to support its validity. Ultimately, however, as was the case with the early developments in quantum mechanics (including Schrödinger’s equation), new theoretical results must be validated (or invalidated) by appropriate empirical evidence.

### Future work

Our results are based on the two-wheeled carriage as the classical model of the molecular wheelbarrow. However, the center of mass assumption—that the car’s center of mass is midway on the line connecting the center of the two wheels—may not be satisfied by real-world nanovehicles. Models of nonholonomic vehicles with off-center center of mass exist, however, and preliminary work indicates that some of these systems are “Chaplygin Hamiltonizable.” These systems are another well-studied class of “Hamiltonian-like” nonholonomic systems^12^ that are also amenable to the quantization approach taken herein.

Our work also relied on a reasonably simple method to derive the potential energy function depicted in Fig. 1(c). This simple modeling of the potential energy function allowed us to capture the effects of nonholonomic constraints in a controlled way, but ignored surface interactions. Future models will need to account for surface interactions in order to build predictive models that can be more directly experimentally tested. Such models may also consider motion on non-flat, chemically realistic surfaces, which may change the symmetry group associated with translational motion.

## Methods

### Mathematical model of the molecular wheelbarrow

#### Classical mechanics

The configuration space of the two-wheeled carriage (Fig. 1(a)) is *Q*_0_ = $SE\mathrm{(2)}\times {T}^{2}$; the system’s Lagrangian and constraints were presented in^8^, which also introduced the angles $\theta =\frac{1}{2}({\psi }_{1}+{\psi }_{2})$ and ${\theta }_{2}=\frac{1}{2}({\psi }_{1}-{\psi }_{2})$ to simplify the presentation. In our context, ${\theta }_{2}=\frac{1}{2}{\theta }_{d}$. In the new coordinates $(\phi ,x,y,\theta ,{\theta }_{d})\in {Q}_{0}$, the system’s Lagrangian and constraints are^8^:

1 $$\begin{array}{l}L=\frac{1}{2}[{I}_{1}{\dot{\phi }}^{2}+I({\dot{\theta }}^{2}+\frac{1}{4}{\dot{\theta }}_{{\rm{d}}}^{2})+m({\dot{x}}^{2}+{\dot{y}}^{2})],\,\dot{x}\,\sin \,\phi -\dot{y}\,\cos \,\phi =0,\\ \,\dot{x}\,\cos \,\phi +\dot{y}\,\sin \,\phi =a\dot{\theta },\,\dot{\phi }=\frac{a}{2w}{\dot{\theta }}_{{\rm{d}}},\end{array}$$

where we have introduced *I* = 2*Iw*. The first constraint expresses the fact that the velocity perpendicular to the wheels is zero; the second relates the velocity of the center of mass in the direction of the wheels to the angular velocity of each wheel. Solving these for $\dot{x}$ and $\dot{y}$ yields $\dot{x}-a(\cos \,\phi )\dot{\theta }=0$ and $\dot{y}-a(\sin \,\phi )\dot{\theta }=0$. The last constraint yields ${\theta }_{{\rm{d}}}(t)=(2w/a)\phi (t)+{\theta }_{{\rm{d}}}(0)$. Without loss of generality we can take ${\theta }_{{\rm{d}}}\mathrm{(0)}=0$. Therefore, ${\theta }_{{\rm{d}}}=\mathrm{(2}w/a)\phi$. Using this in *L* and introducing $J\,:\,={I}_{1}+I{(w/a)}^{2}$ yields

2 $$L=\frac{1}{2}[J{\dot{\phi }}^{2}+I{\dot{\theta }}^{2}+m({\dot{x}}^{2}+{\dot{y}}^{2})],\,\dot{x}=a(\cos \,\phi )\dot{\theta },\,\dot{y}=a(\sin \,\phi )\dot{\theta }\mathrm{.}$$

The resulting configuration space is ${Q}_{1}={S}^{1}\times {S}^{1}\times {{\mathbb{R}}}^{2}$ and the equations of motion are^13^ [Sect. 5.6.1]:

3 $$J\ddot{\phi }=\mathrm{0,}\,(I+m{a}^{2})\ddot{\theta }=\mathrm{0,}\,\dot{x}=a(\cos \,\phi )\dot{\theta },\,\dot{y}=a(\sin \,\phi )\dot{\theta }\mathrm{.}$$

If $\dot{\phi }\mathrm{(0)}=0$ the resulting trajectory is a straight line, *φ* = *φ*_0_, the constraints in (2) are integrable, and the system is *holonomic* (see Section E of the Appendix). When $\dot{\phi }\mathrm{(0)}\ne 0$, the constraints in (2) are non-integrable, the system is nonholonomic, and the wheelbarrow moves in circular trajectories centered at (*x*_0_, *y*_0_) of radius $a\dot{\theta }\mathrm{(0)/}\dot{\phi }\mathrm{(0)}$. (This confirms the results in^8^).

#### Modeling of the molecular wheelbarrow

Modeling a molecule’s potential energy as a function of its nuclear coordinates upon conformational change (here, relative rotation of the wheels) is made simpler if the system is electronically adiabatic and electronic degrees of freedom respond “instantaneously” to nuclear motions. In such cases, electronic energies can be represented as a function of nuclear coordinates; this simplification is known as the Born-Oppenheimer approximation^14^. As the axle in the molecular wheelbarrow in^5^ includes carbon-carbon single bonds, intramolecular rotation may be possible via electronically adiabatic processes, and so employing the Born-Oppenheimer approximation to model the potential energy function of the molecular wheelbarrow in^5^ is reasonable.

To generate a coarse model of the potential energy curve, *ab initio* calculations were performed on the molecule using the Gaussview/Gaussian (Gaussian, Inc., Wallingford, CT) and the ORCA^10^ software packages. The true potential energy of the experimental system would include both changes in intramolecular interactions upon relative wheel rotation and interactions with the surface, but here we ignore the surface interaction (for the reasons mentioned in the Results section). Electronic energies were computed as a function of the dihedral angle (${\theta }_{{\rm{d}}}=\mathrm{(2}w/a)\phi$ in our chosen variables) shown in Fig. 1(b), sampled at 30-degree increments. At each fixed dihedral value, all other internal degrees of freedom were optimized and the resulting energy evaluated using the B3LYP hybrid density functional method^15,16^ with the D3 dispersion correction^17^ and the 6-31G^*^ basis set. The experimental synthetic scheme used^9^ produced a mixture of the meso and chiral diastereomers of the molecule. In the meso compound (shown in Fig. 1(b)), one wheel is the mirror image of the other, such that the nitrogen tags line up at a dihedral angle of zero, while in the R/R and S/S chiral compounds, they do not, and the molecule is in the symmetry point group C_2_. We modeled both the meso and chiral stereoisomers, and the resulting potential energy curves (with the zero of energy set to the minimum of each curve) are shown in Fig. 1(c).

As expected, the potential energy curve of the meso isomer is symmetric about dihedral angles of 0 and 180 degrees, while the curve of the chiral isomer is not. Both potential energy curves nevertheless reveal a very modest well depth of ~0.3 kJ/mol (3 meV), with a maximum roughly corresponding to the angle at which the nitrogen atoms are within closest proximity. While a barrier of 0.3 kJ/mol is fairly negligible at room temperature, it is significant at the experimental temperature of 5 K, at which the thermal energy is roughly 0.04 kJ/mol (0.4 meV). The reason for the barrier is likely electrostatic repulsions between the partially negatively charged nitrogen tags. To test this hypothesis, we created a model of the achiral variant of the wheelbarrow in which the nitrogen tags were replaced by CH moieties. The resulting potential energy curve (shown on the same graph) had a periodicity expected from molecular symmetry, with significantly lower well depths of ~0.05 kJ/mol (0.5 meV), thermally accessible even at 5 K. This result therefore supports our hypothesis that the nitrogen tags are the cause of the barrier. It also suggests that while not achieving the *α*-value needed to enable detection of the negative term in ${\tilde{E}}_{\mathrm{(0,0)}}$, the molecular variant lacking the tags could be closer to an experimental model for relative rotation without a potential barrier (treated in the Special Cases of Interest subsection of the Results section), if one neglects potential energy contributions due to surface interactions.

Figure 1(c) suggests that the molecular wheelbarrow can be roughly modeled by (2) with an added pendulum potential:

4 $$V(\phi )=\alpha \mathrm{[1}+\,\cos \,\mathrm{(2}w\phi /a)],$$

where *α* determines the depth of the potential well. We stress that (4) expresses *V* as a function of *φ* through ${\theta }_{{\rm{d}}}=2w\phi /a$, an outcome of the nonholonomic system data in (1). Moreover, adding (4) to our model forces the classical wheelbarrow’s trajectories to be non-linear, since *θ*_d_ measures the wheels’ relative rotation. The potential (4) is nearly equivalent to the standard pendulum potential *α*(1 − cos*φ*). (As we discuss later, 2*w*/*a* ≈ 1 for the molecular wheelbarrow we apply our results to, and the change of variables $\phi \mapsto \phi +\pi$ converts 1 − cos*φ* to 1 + cos*φ*.) Our final mathematical model is therefore:

5 $${L}_{{\rm{mw}}}=\frac{1}{2}[J{\dot{\phi }}^{2}+I{\dot{\theta }}^{2}+m({\dot{x}}^{2}+{\dot{y}}^{2})]-\alpha \mathrm{[1}+\,\cos \,\mathrm{(2}w\phi /a)],\,\dot{x}=a(\cos \,\phi )\dot{\theta },\,\dot{y}=a(\sin \,\phi )\dot{\theta }\mathrm{.}$$

The corresponding (classical) equations of motion are

6 $$J\ddot{\phi }=\mathrm{(2}w\alpha /a)\,\sin \,\mathrm{(2}w\phi /a),\,(I+m{a}^{2})\ddot{\phi }=\mathrm{0,}\,\dot{x}=a(\cos \,\phi )\dot{\theta },\,\dot{y}=a(\sin \,\phi )\dot{\theta }\mathrm{.}$$

### Quantization of the model

The main barrier to quantizing the system (5) is that it is a nonholonomic system (Section A in the Supplementary Appendix reviews these systems) and nonholonomic systems are not Hamiltonian systems^13^. Therefore the standard quantization procedure cannot be applied to quantize nonholonomic systems. Past attempts at overcoming this hurdle have “resorted to a variety of *ad hoc* techniques that have generally led to poor results”^7^ (see^7^ [Section 4] for a brief history of such attempts). However, in^7^ a class of nonholonomic systems known as *conditionally variational systems* was successfully quantized using geometric quantization. Conditionally variational systems are nonholonomic systems whose equations of motion are reproduced exactly by the Hamiltonian mechanics of a Hamiltonian system whose initial conditions satisfy the nonholonomic constraints. The remainder of this section leverages these results as follows. First, we employ the results of^18^ to recast (5) as a conditionally variational system. Then, we quantize the resulting system by modifying the approach of^7^. (That approach needs modification because^7^ only considered Euclidean configuration spaces, and the configuration space of (5) is the non-Euclidean space *Q*_1_.)

#### Quantizable representation of the molecular wheelbarrow model

The system (5) is equivalent to the “vertically rolling disk” system subject to the potential (4). The vertically rolling disk system is a well-known nonholonomic system^13^ [Sect. 5.6.1] whose dynamics are invariant under translations in the plane. (Such systems are called *abelian Chaplygin* nonholonomic systems; Section A in the Supplementary Appendix reviews these systems.) The associated action of the Lie group ${G}_{1}={{\mathbb{R}}}^{2}$ on the system’s configuration space *Q*_1_ yields the reduced configuration space ${\bar{Q}}_{1}={Q}_{1}/{G}_{1}={S}^{1}\times {S}^{1}$; the local coordinates of ${\bar{Q}}_{1}$ are (*r*^*a*^) = (*θ*, *φ*), and those of *G*_1_ are (*s*^*a*^) = (*x*, *y*).

In^18^, the vertically rolling disk system was shown to be a conditionally variational system. Since the nonholonomic system defining the molecular wheelbarrow (given by the data (5)) is equivalent to the vertically rolling disk system with the potential (4), and since that potential is only a function of the coordinates, Part (3) of^18^ [Proposition 3] applies and it follows that the system (5) is also conditionally variational. Equation (3.13) in^18^ can then be used to calculate the Lagrangian of the aforementioned associated Hamiltonian system. The resulting kinetic energy metric is not positive definite, a requirement of the quantization we undertake in the next subsection, but the following modified Lagrangian resolves that issue:

7 $${L}_{{\rm{hmw}}}=\frac{1}{2}[J{\dot{\phi }}^{2}+(I+2m{a}^{2}){\dot{\theta }}^{2}+m({\dot{x}}^{2}+{\dot{y}}^{2})]-ma\dot{\theta }(\dot{x}\,\cos \,\phi +\dot{y}\,\sin \,\phi )-\alpha \mathrm{[1}+\,\cos \,\mathrm{(2}w\phi /a\mathrm{)].}$$

Here “hmw” reminds the reader that (7) is the Lagrangian of the Hamiltonian system whose Hamiltonian mechanics reproduce (6) when the initial conditions satisfy the nonholonomic constraints in (5). The Hamiltonian associated with (7) is

8 $$\begin{array}{rcl}{H}_{{\rm{hmw}}} & = & \frac{1}{2\beta }[\frac{\beta }{J}{p}_{\phi }^{2}+{p}_{\theta }^{2}+\frac{\beta }{m}({p}_{x}^{2}+{p}_{y}^{2})+a({p}_{x}\,\cos \,\phi +{p}_{y}\,\sin \,\phi )\\  &  & \times \,(a{p}_{x}\,\cos \,\phi +a{p}_{y}\,\sin \,\phi +2{p}_{\theta })\phantom{\frac{1}{0}}\,]+\alpha \mathrm{[1}+\,\cos \,\mathrm{(2}w\phi /a)],\end{array}$$

where *β* = *I* + *ma*^2^ > 0. Now, ${p}_{x}=m(\dot{x}-a(\cos \,\phi )\dot{\theta })$ and ${p}_{y}=m(\dot{y}-a(\sin \,\phi )\dot{\theta })$ are the *x*- and *y*-conjugate momenta, respectively, calculated from (7). Since (8) is independent of *x* and *y*, then ${\dot{p}}_{x}=0$ and ${\dot{p}}_{y}=0$, so that ${p}_{x}(t)={p}_{x}(t=\mathrm{0)}=\,:{\mu }_{x}$ and ${p}_{y}(t)={p}_{y}(t=\mathrm{0)}=\,:{\mu }_{y}$, where ${\mu }_{i}\in {\mathbb{R}}$ are constants. The Euler-Lagrange equations of (7) are:

9 $$\begin{array}{rcl}J\ddot{\phi } & = & \mathrm{(2}\alpha w/a)\,\sin \,\mathrm{(2}w\phi /a),\,(I+m{a}^{2})\ddot{\theta }=a({\mu }_{{y}}\,\cos \,\phi -{\mu }_{{x}}\,\sin \,\phi )\dot{\phi },\\ \dot{x} & = & a(\cos \,\phi )\dot{\theta }+\frac{{\mu }_{x}}{m},\,\dot{y}=a(\sin \,\phi )\dot{\theta }+\frac{{\mu }_{y}}{m}\mathrm{.}\end{array}$$

Therefore, if the initial conditions $({\phi }_{0},\,{\theta }_{0},\,{x}_{0},\,{y}_{0},\,{\dot{\phi }}_{0},\,{\dot{\theta }}_{0},\,{\dot{x}}_{0},\,{\dot{y}}_{0})$ satisfy the nonholonomic constraints in (5), then ${\mu }_{x}={p}_{x}(t=\mathrm{0)}=0$ and ${\mu }_{y}={p}_{y}(t=\mathrm{0)}=0$, and (9) reproduces (6). Importantly, this shows that the nonholonomic constraints in (5) are enforced in (9) by setting the initial values of *p*_*x*_ and *p*_*y*_ (i.e., *μ*_*x*_ and *μ*_*y*_, respectively) equal to zero.

#### Verifying pre-quantization requirements

We now quantize the Hamiltonian system generated by *H*_hmw_—which we will refer to via the pair (*Q*_1_, *H*_hmw_)—and, following^7^, enforce the nonholonomic constraints at the quantum level by choosing suitable initial conditions akin to setting *μ*_*x*_ = *μ*_*y*_ = 0. Because *Q*_1_ is not Euclidean, we will employ geometric quantization. (An excellent and short review of the subject is given in^19^, with more comprehensive treatments to be found in^20–23^.) This requires verifying the following requirements first.

1. *Q*_1_ *is connected and orientable, and also a smooth Riemannian manifold with respect to the kinetic energy metric of L*_*hmw*_. Since ${Q}_{1}={S}^{1}\times {S}^{1}\times {{\mathbb{R}}}^{2}$ is the Cartesian product of connected and orientable smooth manifolds, it is itself a connected and orientable smooth manifold. Now, the kinetic energy metric of (7) is
  10 $${g}_{{\rm{hmw}}}=(\begin{array}{cccc}J & 0 & 0 & 0\\ 0 & I+2m{a}^{2} & -ma\,\cos \,\phi  & -ma\,\sin \,\phi \\ 0 & -ma\,\cos \,\phi  & m & 0\\ 0 & -ma\,\sin \,\phi  & 0 & m\end{array})\mathrm{.}$$
  A straightforward calculation shows that all of the determinants of the upper-left *l* × *l* submatrices (where $l=\mathrm{1,}\,\ldots ,\,4$) of (10) are positive. It follows from Sylvester’s criterion^24^ [Theorem 7.2.5] that *g*_hmw_ is positive definite. Therefore, it is a positive definite symmetric (0, 2)-tensor, which makes it a Riemannian metric on *Q*_1_.
2. *Q*_1_ *is complete with respect to the metric induced by g*_hmw_. We start with^25^ [Prop. 7.2.5], which shows that a Riemannian manifold’s Riemannian metric *g* induces a metric space structure on the manifold, provide the manifold is connected. The induced distance^25^ [Section 7.2] is the infimum of
  11 $${L}_{g}(\gamma ):\,={\int }_{a}^{b}\sqrt{g(\gamma ^{\prime} (t),\gamma ^{\prime} (t))}\,dt,$$
  the length of a piecewise differentiable path connecting *p* and *q*, where *p*, *q* ∈ *Q* and *γ*: [*a*, *b*] → *Q*. Applying this result here to *Q*_1_ and the metric *g*_hmw_ yields the metric space $({Q}_{1},\,{d}_{{g}_{{\rm{hmw}}}})$.
  We now prove that *Q*_1_ is complete with respect to the metric induced by *g*_hmw_. First, we fix *q* ∈ *Q* and let
$v\in {T}_{q}{Q}_{1}\cong {{\mathbb{R}}}^{4}$. Then *g*_hmw_(*v*, *v*) is a quadratic form we denote by *f*: $f(v)={g}_{{\rm{hmw}}}(v,\,v)={v}^{T}Mv$, where *M* is the matrix (10) and $v=({v}_{1},\,\ldots ,\,{v}_{4})$, with *v*_*i*_ the *i*-th component of the vector v (^26^ [Section V.7]). We now follow the proof of^7^ [Theorem 3]. Namely, since *g*_hmw_ is a Riemannian metric, *M* is a positive-definite and symmetric matrix. It follows from the Principal Axes Theorem^27^ [Chapter X, Theorem 19] that *M* is orthogonally diagonalizable, that is, there is an orthogonal matrix *O* such that *O*^*T*^*MO* = *D*, where *D* is the diagonal matrix of eigenvalues of *M*. As a consequence, if ${\lambda }_{1}(q),\,\ldots ,\,{\lambda }_{4}(q)$ are the eigenvalues of *M* then in the new variable *y* = *O*^*T*^*v* (or *v* = *Oy*) we have $f(Oy)={g}_{{\rm{hmw}}}(Oy,\,Oy)={y}^{T}{O}^{T}MOy={y}^{T}Dy={\lambda }_{i}(q){y}_{i}^{2}\mathrm{.}$ In our present setting, the eigenvalues of (10) are
  12 $${\lambda }_{1}=J,\,{\lambda }_{2}=m,\,{\lambda }_{\mathrm{3,4}}=\frac{I+m\mathrm{(2}{a}^{2}+\mathrm{1)}\pm \sqrt{{(I-m)}^{2}+4m{a}^{2}(I+m{a}^{2})}}{2}\mathrm{.}$$
  The first two, along with *λ*_3_ (the positive root eigenvalue) are positive since all parameters are assumed positive; a simple calculation shows that *λ*_4_ > 0 if *mβ* > 0, which is true. Now, introducing *b*_1_ = min{*λ*_*i*_} and *b*_2_ = max{*λ*_*i*_},
  $$\begin{array}{c}{b}_{1}({y}_{1}^{2}+\ldots +{y}_{4}^{2})\le f(Oy)\le {b}_{2}({y}_{1}^{2}+\ldots +{y}_{4}^{2})\\ \,\iff \,{b}_{1}{y}^{T}y\le f(Oy)\le {b}_{2}{y}^{T}y\\ \,\iff \,{b}_{1}{v}^{T}v\le f(v)\le {b}_{2}{v}^{T}v,\end{array}$$
  since *v* = *Oy*. Denoting the norm of *v* with respect to the Euclidean metric *g*_*e*_ on *Q*_1_ by ||*v*||_*e*_, the previous inequality becomes
  13 $${b}_{1}||v{||}_{e}^{2}\le f(v)\le {b}_{2}||v{||}_{e}^{2}\mathrm{.}$$
  This is true for all *q* ∈ *Q*_1_ since *b*_1_ and *b*_2_ are independent of *q* and *q* ∈ *Q*_1_ was arbitrary. Following again the proof of^7^ [Theorem 3], if we denote by $${d}_{{g}_{e}}$$ the distance induced by the Riemannian metric *g*_*e*_ on $${T}_{q}{Q}_{1}\cong {{\mathbb{R}}}^{4}$$ (the usual Pythagorean distance), then using (13) in (11) implies that $${b}_{1}{d}_{{g}_{e}}(p,\,q)\le {d}_{{g}_{{\rm{hmw}}}}$$$$(p,\,q)\le {b}_{2}{d}_{{g}_{e}}(p,\,q)$$ for any *p*, *q* ∈ *Q*_1_. Thus, every Cauchy sequence in the metric space $$({Q}_{1},\,{d}_{{g}_{{\rm{hmw}}}})$$ is also a Cauchy sequence in the metric space $$({Q}_{1},\,{d}_{{g}_{e}})$$. In our present setting, $$({Q}_{1},\,{d}_{{g}_{e}})$$ is complete—it is the product of the complete spaces (*S*^1^ × *S*^1^, *d*_1_) and $$({{\mathbb{R}}}^{2},\,{d}_{2})$$, where *d*_1_ and *d*_2_ are Euclidean metrics and $${d}_{{g}_{e}}$$ is the product metric: $${d}_{{g}_{e}}(p,\,r)=(({d}_{1}({p}_{1},\,{p}_{2}{))}^{2}+$$$${{({d}_{2}({r}_{1},{r}_{2}))}^{2})}^{\mathrm{1/2}}$$—so it follows that $$({Q}_{1},\,{d}_{{g}_{{\rm{hmw}}}})$$ is also complete.
3. *The Hamiltonian vector field* ${X}_{{H}_{{\rm{hmw}}}}$ *is a complete vector field*. We have just shown that $({Q}_{1},{d}_{{g}_{{\rm{hmw}}}})$ is a complete Riemannian manifold. In addition, *V* ≥ 0 in our model (from (4)). Thus, the two assumptions from part (ii) of the theorem in^28^ are satisfied, and it follows from that theorem that ${X}_{{H}_{hmw}}$ is a complete vector field.

#### Quantizing the system

Having verified all requirements to employ geometric quantization, we now apply the following results to the quantize the Hamiltonian system (*Q*_1_, *H*_hmw_) in the Schrödinger representation.

1. *The wave functions and Hilbert space*. The wave functions $\psi (q)\in {C}^{\infty }({Q}_{1},{\mathbb{C}})$ (i.e., they are complex-valued differentiable functions of *q* ∈ *Q*_1_)^20,21^; the Hilbert space $H\cong {L}^{2}({Q}_{1},\,\sqrt{{\rm{\det }}\,{g}_{{\rm{hmw}}}})$, the space of complex-valued functions on *Q*_1_ that are square integrable with respect to the density $\sqrt{{\rm{\det }}\,{g}_{{\rm{hmw}}}}$ (see^20,22)^.
2. *The quantum operators*. For an observable *f* at most linear in the momenta (i.e., $f(q,\,p)={a}_{0}(q)+{a}_{i}(q){p}_{i}$), its quantum operator—denoted hereafter by $\hat{f}$—is given by^20,22^
  14 $$\hat{f}=-\,i\hslash {a}_{i}(q)\frac{\partial }{\partial {q}^{i}}+{a}_{0}(q)-\frac{1}{2}i\hslash \sum _{i\mathrm{=1}}^{n}\frac{\partial {a}_{i}}{\partial {q}^{i}}\mathrm{.}$$
  It follows from this that ${\hat{q}}^{i}={q}^{i}$ and ${\hat{p}}_{i}=-\,i\hslash \partial /\partial {q}^{i}$ (the standard operators).
3. *The Hamiltonian operator*. The Hamiltonian operator ${\hat{H}}_{{\rm{hmw}}}$, calculated in^22^ [Sec. 9.7] and^23^ [Chapter 9], is given by:

15 $${\hat{H}}_{{\rm{hmw}}}=-\,\frac{{\hslash }^{2}}{2}({\rm{\Delta }}-\frac{R}{6})+V,$$

where Δ is the Laplace-Beltrami operator, *R* is the Ricci scalar curvature of *g*_hmw_, *V* the potential (4).

We begin with the observation that *H*_hmw_ is independent of *x* and *y*, so the operators ${\hat{p}}_{x}$ and ${\hat{p}}_{y}$ commute with ${\hat{H}}_{{\rm{hmw}}}$, and therefore they share a basis of simultaneous eigenfunctions. The eigenfunction equations for ${\hat{p}}_{x}$ and ${\hat{p}}_{y}$ are

16 $${\hat{p}}_{x}(\psi )={\mu }_{x}\psi ,\,{\hat{p}}_{y}(\psi )={\mu }_{y}\psi \Rightarrow -\,i\hslash \frac{\partial \psi }{\partial x}={\mu }_{x}\psi ,\,-\,i\hslash \frac{\partial \psi }{\partial y}={\mu }_{y}\psi \mathrm{.}$$

These yield $\psi (\theta ,\,\phi ,\,x,\,y)={\psi }_{r}(\theta ,\,\phi ){e}^{\frac{i}{\hslash }({\mu }_{x}x+{\mu }_{y}y)}$. Next, we used Mathematica and the formula for *R* (see^25^) to calculate that

17 $$R=-\,\frac{m{a}^{2}}{2\beta J},$$

where we recall that *β* = *I* + *ma*^2^. The resulting time-independent Schrödinger equation ${\hat{H}}_{{\rm{hmw}}}(\psi )=E\psi$ is a complicated equation (c.f. formula (B.1) in Section B of the Supplemental Appendix) with (to our knowledge) no known analytical solution. However, we have yet to enforce the classical nonholonomic constraints at the quantum level. Recall that the Hamiltonian system (*Q*_1_, *H*_hmw_) only reproduces the nonholonomic mechanics of the wheelbarrow when *μ*_*x*_ = *μ*_*y*_ = 0, which corresponds to a particular choice of initial conditions (namely, ones that satisfy the nonholonomic constraints). Enforcing those nonholonomic constraints at the quantum level amounts to choosing a similarly particular choice of initial condition—the particular initial time-dependent wave function ${{\rm{\Psi }}}_{0}(q)\,:\,={\rm{\Psi }}(q,\,t=\mathrm{0)}$ satisfying

18 $$0={\langle {\hat{p}}_{a}\rangle }_{{{\rm{\Psi }}}_{0}}=\langle {{\rm{\Psi }}}_{0}(q),{\hat{p}}_{a}({{\rm{\Psi }}}_{0}(q))\rangle =-\,i\hslash \int {{\rm{\Psi }}}_{0}(q)[\frac{\partial \overline{{{\rm{\Psi }}}_{0}}(q)}{\partial {s}^{a}}]\sqrt{{\rm{\det }}\,{g}_{{\rm{hmw}}}}\,{d}^{4}q,$$

where $q=(\phi ,\,\theta ,\,x,\,y)$, (*s*^*a*^) = (*x*, *y*), and $\sqrt{{\rm{\det }}\,{g}_{{\rm{hmw}}}}=m\sqrt{\beta J}$. (Note that any Ψ_0_(*q*) independent of *x* and *y* will satisfy (18).) Thus, in the classical setting, imposing *μ*_*x*_ = *μ*_*y*_ = 0 enforces the nonholonomic constraints, while in the quantum setting, such a choice corresponds to only imposing the nonholonomic constraints *on average*.

Supposing initial wave functions are chosen as in (18), ${\hat{H}}_{{\rm{hmw}}}(\psi )=E\psi$ simplifies considerably to

19 $$\beta \frac{{\partial }^{2}{\psi }_{r}}{\partial {\phi }^{2}}+J\frac{{\partial }^{2}{\psi }_{r}}{\partial {\theta }^{2}}=-\,\frac{2\beta J}{{\hslash }^{2}}[\tilde{E}+\frac{m{a}^{2}{\hslash }^{2}}{24\beta J}-\alpha \mathrm{[1}+\,\cos \,\mathrm{(2}w\phi /a)]]{\psi }_{r},$$

where $\tilde{E}$ is now the energy corresponding to the *p*_*x*_ = *p*_*y*_ = 0 case of (8). Equation (19) is separable, and substituting in ${\psi }_{r}(\phi ,\theta )=\chi (\theta )\rho (\phi )$ and using the separation constant *λ* gives the two equations

20 $$\tfrac{{\partial }^{2}\chi }{\partial {\theta }^{2}}+(\tfrac{2J}{{\hslash }^{2}}\hat{E}-\tfrac{\lambda }{\beta })\chi =0,\,\tfrac{{\partial }^{2}\rho }{\partial {\phi }^{2}}+\tfrac{2\beta }{{\hslash }^{2}}[\tfrac{\lambda {\hslash }^{2}}{2\beta J}-\alpha \mathrm{[1}+\,\cos \,\mathrm{(2}w\phi /a)]]\rho =\mathrm{0,}\,\hat{E}=\tilde{E}+\tfrac{m{a}^{2}{\hslash }^{2}}{24\beta J}\mathrm{.}$$

The periodic boundary condition $\chi (\theta )=\chi (\theta +2\pi )$ implies that the first equation (20) has the general solutions ${\chi }_{k}(\theta )={c}_{1}\,\sin (k\theta )+{c}_{2}\,\cos (k\theta )$ for each $k\in {\mathbb{Z}}$, where ${k}^{2}=2J\widehat{E}/{\hslash }^{2}-\lambda /\beta$. We can rewrite these general solutions as

21 $${\chi }_{k}(\theta )=C\,\cos (k\theta -\delta ),\,{\hat{E}}_{(k)}=\frac{{\hslash }^{2}{k}^{2}}{2J}+\frac{\lambda {\hslash }^{2}}{2\beta J},\,k\in {\mathbb{Z}},$$

where *C* and *δ* depend on *c*_1_ and *c*_2_, and are constants determined ultimately by the normalization of the eigenfunctions. Turning our attention now to the second equation in (20), we note that this is formally the equation of a quantum pendulum^29^. The periodic boundary condition is now $\rho (\phi )=\rho (\phi +2\pi )$, and after the change of variable $\phi =az/w$, the equation becomes

22 $$\frac{{{\rm{\partial }}}^{2}\rho }{{\rm{\partial }}{z}^{2}}+[A-2\gamma \,\cos \,(2z)]\rho =0,\,A=\frac{2{a}^{2}\beta }{{w}^{2}{\hslash }^{2}}({\hat{E}}_{(k)}-\frac{{\hslash }^{2}{k}^{2}}{2J}-\alpha ),\,\gamma =\frac{{a}^{2}\alpha \beta }{{w}^{2}{\hslash }^{2}},$$

which is a classic Mathieu equation^30^. Now, since $\rho \mathrm{(2}z)=\rho \mathrm{(2(}z+\pi ))$, *ρ* is *π*-periodic as a function of *z*. Such solutions to (22) are known as the *angular Mathieu functions of even order*, and are denoted by *ce*_2n_(*z*, *γ*) and *se*_2*n*+2_(*z*, *γ*) (the Mathieu cosine and sine functions, respectively), where *n* = 0, 1, …. Thus, the general solutions to the second equation in (20) are:

23 $${\rho }_{n}(\phi )={A}_{n}c{e}_{2n}(\frac{\phi }{2},\gamma )+{B}_{n}s{e}_{2n+2}(\frac{\phi }{2},\gamma ),\,n=\mathrm{0,}\,\mathrm{1,}\,\ldots $$

Denoting by *a*_2*n*_(*γ*) and *b*_2*n*+2_(*γ*) the corresponding eigenvalues of the Mathieu cosine and sine functions, respectively, it then follows from (22) that the *φ*-energies associated with the angular Mathieu cosine and sine functions are

24 $${\hat{E}}_{(k,n)}(\gamma )=\alpha +\frac{{\hslash }^{2}{k}^{2}}{2J}+\frac{{w}^{2}{\hslash }^{2}}{2{a}^{2}\beta }{a}_{2n}(\gamma ),\,{\hat{E}}_{(k,n)}(\gamma )=\alpha +\frac{{\hslash }^{2}{k}^{2}}{2J}+\frac{{w}^{2}{\hslash }^{2}}{2{a}^{2}\beta }{b}_{2n+2}(\gamma ),\,n=0,\,1,\,\ldots ,$$

respectively. Since the eigenvalues of the angular Mathieu functions are strictly ordered, introducing

$${c}_{n}(\gamma )=\{\begin{array}{ll}{a}_{n}(\gamma ), & n=2m\\ {b}_{n+1}(\gamma ), & n=2m+1\end{array},\,m,\,n=\mathrm{0,}\,\mathrm{1,}\,\mathrm{2,}\ldots $$

allows us to combine (24) into one equation:

25 $${\widehat{E}}_{(k,n)}(\gamma )=\alpha +\frac{{\hslash }^{2}{k}^{2}}{2J}+\frac{{w}^{2}{\hslash }^{2}}{2{a}^{2}\beta }{c}_{n}(\gamma ),\,n=\mathrm{0,}\,\mathrm{1,}\,\ldots $$

Finally, substituting (25) into the third equation in (20) yields the energy spectrum of (19):

26 $${\tilde{E}}_{(k,n)}=\alpha +\frac{{\hslash }^{2}{k}^{2}}{2J}+\frac{{w}^{2}{\hslash }^{2}}{2{a}^{2}\beta }{c}_{n}(\gamma )-\frac{m{a}^{2}{\hslash }^{2}}{24\beta J},\,\gamma =\frac{{a}^{2}\alpha \beta }{{w}^{2}{\hslash }^{2}},\,k\in {\mathbb{Z}},\,n=\mathrm{0,}\,\mathrm{1,}\,\ldots $$

When *γ* is large (we later show that this is true of the molecular wheelbarrow we apply our results to) and for not too large *n*, *a*_2*n*_(*γ*) and *b*_2*n*+2_(*γ*) have the asymptotic approximations^30^ [Chapter XI]:

27 $${a}_{2n}(\gamma )\approx -\,2\gamma +\mathrm{(8}n+\mathrm{2)}\sqrt{\gamma },\,{b}_{2n+2}(\gamma )\approx -\,2\gamma +\mathrm{(8}n+\mathrm{6)}\sqrt{\gamma }\mathrm{.}$$

Using the *a*_0_(*γ*) expression in (26), it follows that the ground state energy (*k* = *n* = 0) is

28 $${\tilde{E}}_{\mathrm{(0,0)}}\approx \frac{w\hslash }{a}\sqrt{\frac{\alpha }{\beta }}-\frac{m{a}^{2}{\hslash }^{2}}{24\beta J}\mathrm{.}$$

Now, from (21, 23) it follows that the full wave function is

29 $${\rm{\Psi }}(q,\,t)=B{\int }_{{{\mathbb{R}}}^{2}}\sum _{k=-\infty }^{\infty }\sum _{n=0}^{\infty }{\varphi }_{1}(x){\varphi }_{2}(y){\chi }_{k}(\theta ){\rho }_{n}(\phi ){e}^{\frac{i}{\hslash }({\mu }_{x}x+{\mu }_{y}y)}{e}^{-i{E}_{(k,n,{\mu }_{x},{\mu }_{y})}t/\hslash }\,dxdy\mathrm{.}$$

The *ϕ*_*i*_ in (29) are determined ultimately by the initial quantum wave function Ψ(*q*, 0) and *B* found through normalization. We note that an application of perturbation theory shows that the full energy of the molecular wheelbarrow ${E}_{(k,n,{\mu }_{x},{\mu }_{y})}$ present in (29) is, to first-order in *μ*_*x*_ and *μ*_*y*_, $\tilde{E}$_(*k*,*n*)_ (c.f., Section C in the Supplemental Appendix).

### Special cases of interest

We have thus far focused on the model (5) of the molecular wheelbarrow. This model includes nonholonomic constraints and the potential (4). This is one of the five variations of the molecular wheelbarrow model considered in the Table 2.

The No Rolling cases describe the dynamics of a *sliding* wheelbarrow (if on a surface), or if not on a surface, a wheelbarrow whose body can rotate in the *xy*-plane but not in the *z*-direction. (Notably, relative wheel rotation does not lead to translational motion here, because the angles *θ*_d_ and *φ* are only related in the rolling case, via the last constraint in (1)). The Nonholonomic cases describe the dynamics of a wheelbarrow rolling on a flat surface subject to the nonholonomic constraints in (5). Such a system moves in circular trajectories, as discussed below (3). The Holonomic case describes the dynamics of a wheelbarrow rolling on a flat surface subject to the constraints (5) in which *φ* = *φ*_0_. As discussed below (3), such a system is holonomic and its trajectories are lines. The prior sections investigated the quantum mechanics of the Nonzero Potential Nonholonomic Case. The remaining variants listed in Table 2 are simpler, special cases of that Case. A straightforward check of the requirements for geometric quantization shows that they are satisfied for all of the other cases in Table 2, and straightforward calculations (see Sections E–F in the Appendix) yield the energy spectra of these cases (labels refer to the categories in Table 1):

30 $${\mathop{E}\limits^{ \sim }}_{(k,l,n)}^{{\rm{n}}{\rm{r}}}=\alpha +\frac{{\hslash }^{2}{k}^{2}}{2{I}_{1}}+\frac{{\hslash }^{2}{l}^{2}}{2I}+\frac{{\hslash }^{2}}{2I}{c}_{n}({\gamma }^{{\rm{n}}{\rm{r}}}),\,{\gamma }^{{\rm{n}}{\rm{r}}}=\frac{I\alpha }{{\hslash }^{2}},\,k,\,l\in {\mathbb{Z}},\,n=0,\,1,\,\ldots ,\,{\mathop{E}\limits^{ \sim }}_{(0,0,0)}^{{\rm{n}}{\rm{r}}}\approx \hslash \sqrt{\frac{\alpha }{I}},$$

31 $${\mathop{E}\limits^{ \sim }}_{(k,l,j)}^{{\rm{n}}{\rm{r}},0}=\frac{{\hslash }^{2}}{2}(\frac{{k}^{2}}{{I}_{1}}+\frac{{l}^{2}}{I}+\frac{4{j}^{2}}{I}),\,k,l,j\in {\mathbb{Z}},\,{\mathop{E}\limits^{ \sim }}_{(0,0,0)}^{{\rm{n}}{\rm{r}},0}=0$$

32 $${\tilde{E}}_{(k,l)}^{\mathrm{nh},0}=\frac{{\hslash }^{2}}{2}(\frac{{k}^{2}}{J}+\frac{{l}^{2}}{\beta })-\frac{m{a}^{2}{\hslash }^{2}}{24\beta J},\,k,\,l\in {\mathbb{Z}},\,{\tilde{E}}_{\mathrm{(0,0)}}^{\mathrm{nh},0}=-\,\frac{m{a}^{2}{\hslash }^{2}}{24\beta J}$$

33 $${\tilde{E}}_{l}^{h,0}=\frac{{\hslash }^{2}{l}^{2}}{2\beta },\,l\in {\mathbb{Z}},\,{\tilde{E}}_{0}^{h,0}=0$$

**Table 2:** Lagrangians, constraints, and configuration spaces Q for five models of the molecular wheelbarrow.

Case	Zero Potential	Nonzero Potential
No Rolling (constraints in (5) removed; *Q* = *Q*_0_):	L (from (1))	$L-\alpha [1+\,\cos \,{\theta }_{d}]\,\,\,\,(L\mathrm{from}(1))$
Rolling, Nonholonomic (constraints in (5) included; *Q* = *Q*_1_):	*L*_mw_ with *α* = 0	*L*_mw_
Rolling, Holonomic (constraints in (5) with *φ* = *φ*_0_ included; *Q* = *Q*_1_):	*L (from (2))*	

### Application to Nickel *et al*.

We first need to estimate our model’s parameters (e.g., moments of inertia)^5^. We used an unconstrained, optimized conformation of the molecule at the Hartree-Fock level, using the 6-31G^*^ basis set to compute moments of inertia and wheel diameter. (These parameters were robust to the diastereomer used.) The estimates yielded

$$m\approx 1\times {10}^{-24}\,{\rm{kg}},\,a\approx 6\times {10}^{-10}\,{\rm{m}},\,2w\approx 7\times {10}^{-10}\,{\rm{m}},\,{I}_{w}\approx 9\times {10}^{-44}\,{\rm{kg}}\,{{\rm{m}}}^{{\rm{2}}},\,{I}_{1}\approx 3\times {10}^{-43}\,{\rm{kg}}\,{{\rm{m}}}^{{\rm{2}}}\mathrm{.}$$

(Note: we display only one significant digit but used more in our calculations.) Using these, we estimate $\beta \approx 7\times {10}^{-43}\,{\rm{kg}}\,{{\rm{m}}}^{{\rm{2}}}$, and using *α* = 0.3 kJ/mol = 3 meV we estimate *γ* (from (22)) to be *γ* ≈ 1 × 10^5^. This large *γ*-value validates our earlier usage of the approximations (27). Moreover, for smaller *n*-values (at most *n* ≈ 500), those approximations yield

34 $${\tilde{E}}_{\mathrm{(0,0)}}^{{\rm{nh}}}\approx \frac{w\hslash }{a}\sqrt{\frac{\alpha }{\beta }}-\frac{m{a}^{2}{\hslash }^{2}}{24\beta J}\approx 1\times {10}^{-5}\,{\rm{eV}}-6\times {10}^{-9}\,{\rm{eV}}\approx 1\times {10}^{-5}\,{\rm{eV}}\mathrm{.}$$

Let us now investigate the other variants of the model listed in Table 2. The energies in the rolling, nonholonomic case with zero potential; and the rolling, holonomic case are

$${\tilde{E}}_{(k,l)}^{\mathrm{nh},0}\approx \mathrm{(1}\times {10}^{-7}){k}^{2}+\mathrm{(5}\times {10}^{-8}){l}^{2}-6\times {10}^{-9}\,{\rm{eV}},\,{\tilde{E}}_{l}^{h,0}\approx \mathrm{(5}\times {10}^{-8}){l}^{2},\,k,l\in {\mathbb{Z}},$$

respectively. In the nonzero potential no rolling case, the new *γ*-value (defined in (30)) is *γ*^nr^ ≈ 8 × 10^3^, and we have

35 $${\tilde{E}}_{\mathrm{(0,0,0)}}^{{\rm{nr}}}\approx 3\times {10}^{-5}\,{\rm{eV}}\mathrm{.}$$

In the zero potential no rolling case, (31) becomes

$${\tilde{E}}_{(k,l,m)}^{\mathrm{nr},0}\approx \mathrm{(1}\times {10}^{-7}){k}^{2}+\mathrm{(2}\times {10}^{-7}){l}^{2}+\mathrm{(8}\times {10}^{-7}){j}^{2}\,{\rm{eV}},\,k,\,l,\,j\in {\mathbb{Z}}.$$

## Electronic supplementary material

Appendix
