# Supplementary material for: The Quantum Mechanics of a Rolling Molecular “Nanocar”
Source: Sci Rep. 2018 Oct 5;8:14878. doi: 10.1038/s41598-018-33023-8 (PMC6173740; doi:10.1038/s41598-018-33023-8)
Supplement: Supplementary file 1 — Appendix [file 41598_2018_33023_MOESM1_ESM.pdf]

Supplementary Information Appendix for: "The Quantum Mechanics of a Rolling Molecular 'Nanocar"'

O.E. Fernandez*
Department of Mathematics
Wellesley College
Wellesley, MA 02482

M.L. Radhakrishnan†
Department of Chemistry
Wellesley College
Wellesley, MA 02482

A Nonholonomic Chaplygin Systems

In brief, nonholonomic systems are mechanical systems with non-integrable velocity constraints (a precise definition will be presented shortly), and nonholonomic *Chaplygin* systems are nonholonomic systems with special (translation) symmetries. These concepts are defined in terms of a "mechanical system" on a smooth manifold.

Definition 1. *Let Q be a smooth n-dimensional Riemannian manifold with (Riemannian) metric g, and suppose that it is also connected and orientable. By a* ***mechanical system on*** *Q we will mean a pair (Q, L), where $L\colon TQ \to \mathbb{R}$ is a regular Lagrangian* ***of mechanical type****: $L = T - V$, where $T\colon TQ \to \mathbb{R}$ is the kinetic energy given by $T(q, \dot{q}) = \frac{1}{2}g_{ij}(q)\dot{q}^i\dot{q}^j$—where $i, j = 1, \ldots, n$ and g_{ij} are the components of g—and $V : Q \to \mathbb{R}$ is a smooth function—the potential energy (we identify V with its lift to TQ).*

We note that we will adhere to the Einstein summation convention for repeated indices throughout.

Let us now add constraints to our mechanical system. Suppose that we now define a *constraint distribution* $\mathcal{D} \subset TQ$ by the one-forms $\{\omega^a\}_{a=1}^k$, $k < n$, as

$$\mathcal{D} = \{v \in TQ \,|\, \omega^a(v) = 0,\ a = 1, \ldots, k\}. \tag{A.1}$$

We will assume that the constraints are linear and homogeneous, so that locally $\omega^a(v) = c^a_j(q)\dot{q}^j$, that the constraints are non-integrable, and that $\mathcal{D}$ has constant rank. Then the triple $(Q, L, \mathcal{D})$ is known as a *nonholonomic mechanical system* [1], or simply a nonholonomic system for short.

Now, suppose that a k-dimensional Lie group G acts on Q such that $\overline{Q} := Q/G$ is a manifold; this happens, for example, if G acts freely and properly on Q. Let $\mathfrak{g}$ be the Lie algebra of G, and ξ_Q the infinitesimal generator on Q corresponding to $\xi \in \mathfrak{g}$. We assume that its lifted action leaves L and $\mathcal{D}$ invariant, and that at each $q \in Q$, the tangent space T_qQ can be decomposed as

$$T_qQ = \mathfrak{g}_Q \oplus \mathcal{D}_q, \quad \text{where} \quad \mathfrak{g}_Q\big|_q = \{\xi_Q(q) \,|\, \xi \in \mathfrak{g}\} \tag{A.2}$$

is the tangent to the orbit through $q \in Q$ [1, Section 2.8]. Then we will call $(Q, L, \mathcal{D}, G)$ a *Chaplygin nonholonomic mechanical system* [1, 2].

Chaplygin systems give rise to a principal bundle $\pi\colon Q \to \overline{Q}$, with principal connection $\mathcal{A}\colon TQ \to \mathfrak{g}$ such that $\ker \mathcal{A} = \mathcal{D}$. This connection can then be used to decompose any tangent vector $v_q \in T_qQ$ into horizontal and vertical parts:

$$v_q = \mathrm{hor}(v_q) + \mathrm{ver}(v_q), \tag{A.3}$$
$$\text{where} \qquad \mathrm{hor}(v_q) = v_q - (\mathcal{A}_q(v_q))_Q(q), \qquad \mathrm{ver}(v_q) = (\mathcal{A}_q(v_q))_Q(q).$$

*ofernand@wellesley.edu
†mradhakr@wellesley.edu

We can now form the reduced velocity phase space TQ/G, and the Lagrangian L induces the reduced Lagrangian $l\colon TQ/G \to \mathbb{R}$ satisfying $L = l \circ \pi_{TQ}$, where $\pi_{TQ}\colon TQ \to TQ/G$ is the standard projection. Furthermore, the decomposition (A.3) gives rise to the *reduced constrained Lagrangian* $l_c\colon T\overline{Q} \to \mathbb{R}$ given by $l_c(r, \dot{r}) := L(q, \mathrm{hor}(\dot{q}))$, where $r = \pi(q)$ and $\dot{r} = T_q\pi(\dot{q})$. Locally, we will write the reduced constrained Lagrangian as

$$l_c(r, \dot{r}) = \frac{1}{2} G_{\alpha\beta}(r)\dot{r}^\alpha \dot{r}^\beta - \overline{V}(r), \tag{A.4}$$

where henceforth Greek indices will range from 1 to $m := \dim \overline{Q} = n - k$, the indices a, b, c will range from 1 to $k = \dim G$, and where $\overline{V}\colon \overline{Q} \to \mathbb{R}$ is defined by $V = \overline{V} \circ \pi$. Since we will be dealing exclusively with the reduced constrained Lagrangian, we will drop the overbar on V henceforth. The $G_{\alpha\beta}$ are the components of the metric on the reduced space $\overline{Q}$ induced by g according to $G_r(v_r, w_r) := g_q(\mathrm{hor}(v_q), \mathrm{hor}(w_q))$, where $r = \pi(q)$.

In our paper we deal exclusively with the well-studied subclass where $G = \mathbb{R}^l \times \mathbb{S}^{k-l}$, where $0 \le l \le k$, and such that L is G-invariant. These are called *abelian Chaplygin* nonholonomic mechanical systems [2]. (These nonholonomic systems have translational symmetry in some of the configuration variables.) We will henceforth refer to these systems simply as "Chaplygin systems." Since L is assumed to be G-invariant, we have that $l = L$. We will therefore denote the corresponding reduced constrained Lagrangian l_c by L_c.

To arrive at the local equations of motion of a Chaplygin system we pick a local trivialization $Q = \overline{Q} \times G$, coordinatized by $q = (r, s)$. The action of G is given by left translation on the second factor; the equations of motion then consist of a system of second-order ordinary differential equations on $\overline{Q}$ (the *reduced system*), together with a system of first-order constraint equations [1]:

$$\frac{d}{dt}\frac{\partial L_c}{\partial \dot{r}^\alpha} - \frac{\partial L_c}{\partial r^\alpha} = -\left(\frac{\partial L}{\partial \dot{s}^a}\right)^* B^a_{\alpha\beta}\dot{r}^\beta, \tag{A.5a}$$

$$\dot{s}^a = -A^a_\alpha(r)\dot{r}^\alpha. \tag{A.5b}$$

Here the star indicates that we have substituted the constraints (A.5b) into (A.5a) *after* differentiation, and

$$B^a_{\alpha\beta} = \frac{\partial A^a_\alpha}{\partial r^\beta} - \frac{\partial A^a_\beta}{\partial r^\alpha} \tag{A.6}$$

are the components of the curvature of A. Since we have assumed that the constraints (A.5b) are non-integrable, it follows that at least one of the components $B^a_{\alpha\beta}$ is nonzero [1].

B The Full Schrödinger Equation

The time-independent Schrödinger equation $\hat{H}_{\mathrm{hmw}}(\psi) = E\psi$ resulting from using equation (15) is:

$$\begin{aligned} &-\frac{\hbar^2}{J}\left[\beta\frac{\partial^2\psi_r}{\partial\varphi^2} + J\frac{\partial^2\psi_r}{\partial\theta^2} + \frac{ma^2}{12}\psi_r\right] + \frac{\psi_r}{m}\left[(\beta + ma^2\cos^2\varphi)\mu_x^2 + (\beta + ma^2\sin^2\varphi)\mu_y^2\right] \\ &\quad - a\hbar\left[(2i)\left(\mu_x\cos\varphi + \mu_y\sin\varphi\right)\frac{\partial\psi_r}{\partial\theta} - \frac{a\mu_x\mu_y}{\hbar}\sin(2\varphi)\psi_r\right] = 2\beta\left[E - \alpha[1 + \cos(2w\varphi/a)]\right]\psi_r, \end{aligned} \tag{B.1}$$

where $\psi(\varphi, \theta, x, y) = \psi_r(\varphi, \theta)e^{\frac{i}{\hbar}(\mu_x x + \mu_y y)}$.

C Employing Perturbation Theory in Calculating the Full Quantum Energy Spectrum

Let us now indicate how the full energy spectrum $E_{(k,n,\mu_x,\mu_y)}$ present in equation (29) in our paper could be calculated. To begin, observe that unlike the energies associated with φ and θ, the analysis in the paper did not yield quantized energies associated with x and y; in other words, the μ_x and μ_y in (B.1) are continuous parameters. We can therefore employ perturbation theory to calculate the corrections to

the energy in equation (29) in our paper when μ_x and μ_y are nonzero. To begin, express $E_{(k,n,\mu_x,\mu_y)}$ as a power series about $(\mu_x, \mu_y) = (0, 0)$:

$$E_{(k,n,\mu_x,\mu_y)} = E_{(k,n)} + \mu_x \left(\frac{\partial E_{(k,n,\mu_x,\mu_y)}}{\partial \mu_x} \right)^* + \mu_y \left(\frac{\partial E_{(k,n,\mu_x,\mu_y)}}{\partial \mu_y} \right)^* + \ldots, \tag{C.1}$$

where the stars indicate that we evaluate terms at $(\mu_x, \mu_y) = (0, 0)$ after the computation of the derivatives. From the Hellman-Feynman theorem [3, Sec. 8.4.1] it follows that

$$\left(\frac{\partial E_{(k,n,\mu_x,\mu_y)}}{\partial \mu_i} \right)^* = \left\langle \psi_{(k,n,\mu_x,\mu_y)}(q), \frac{\partial}{\partial \mu_i} \left[\hat{H}_{\mathrm{hmw}}(\psi_{(k,n,\mu_x,\mu_y)}(q)) \right] \right\rangle^*, \qquad i = x, y.$$

Now, from (B.1) it follows that the only possibly nonzero contribution from these inner products are from the terms

$$\left\langle \psi_{(k,n,\mu_x,\mu_y)}(q), -\frac{ai\hbar}{\beta} \left\{ \begin{array}{c} \cos\varphi \\ \sin\varphi \end{array} \right\} \frac{\partial}{\partial \theta}(\psi_{(k,n,\mu_x,\mu_y)}(q)) \right\rangle^*,$$

where $\cos\varphi$ results from differentiation in μ_x and $\sin\varphi$ from differentiation in μ_y. But these inner products contain the integral

$$\int_0^{2\pi} \chi_k(\theta)\chi_k'(\theta)\, d\theta = -\int_0^{2\pi} k\cos(k\theta - \delta)\sin(k\theta - \delta)\, d\theta,$$

which is zero since $k \in \mathbb{Z}$. We conclude that the full energy of the molecular wheelbarrow is, to first-order in μ_x and μ_y, $E_{(k,n)}$.

D Quantization of the Reduced System

Recall that $\overline{Q}_1 = S^1 \times S^1$. The reduced Lagrangian $L_c : T\overline{Q}_1 \to \mathbb{R}$ obtained by substituting the nonholonomic constraints into the Lagrangian in equation (5) of the paper is:

$$L_{\mathrm{c}} = \frac{1}{2} \left[J\dot{\varphi}^2 + \beta\dot{\theta}^2 \right] - \alpha[1 + \cos(2w\varphi/a)].$$

The kinetic energy metric here is $g_{\mathrm{c}} = \mathrm{diag}\{J, \beta\}$, and so all three pre-quantization requirements outlined in our article are trivially satisfied. The Hamiltonian operator defined by equation (15) in the article becomes:

$$\hat{H}_{\mathrm{c}} = -\frac{\hbar^2}{2} \left[\frac{1}{J}\frac{\partial^2}{\partial\varphi^2} + \frac{1}{\beta}\frac{\partial^2}{\partial\theta^2} \right] + \alpha[1 + \cos(2w\varphi/a)].$$

We note that $R = 0$ (since the components of g_c are constant). The corresponding time-independent Schrödinger equation $\hat{H}_{\mathrm{c}}(\psi_r) = \widetilde{E}\psi_r$ is:

$$-\frac{\hbar^2}{2} \left[\frac{1}{J}\frac{\partial^2\psi_r}{\partial\varphi^2} + \frac{1}{\beta}\frac{\partial^2\psi_r}{\partial\theta^2} \right] + \alpha[1 + \cos(2w\varphi/a)]\psi_r = \widetilde{E}\psi_r.$$

This rearranges to equation (19) in our article without the $ma^2\hbar^2/(24\beta J)$ term (this is the R-correction, which is zero this time around). As such, all subsequent results are the same, except for the omission of $ma^2\hbar^2/(24\beta J)$ in all formulas.

E Quantization of the Holonomic System

When $\dot{\varphi}(0) = 0$, $\varphi(t) = \varphi_0$, as explained below equation (3) in our article. Thus, the trajectories of the classical motion of the wheelbarrow are lines. The Lagrangian and constraints then become:

$$L = \frac{1}{2} \left[J\dot{\varphi}^2 + I\dot{\theta}^2 + m(\dot{x}^2 + \dot{y}^2) \right], \quad \dot{x} = a(\cos\varphi_0)\dot{\theta}, \quad \dot{y} = a(\sin\varphi_0)\dot{\theta}. \tag{E.1}$$

Because the constraints are integrable,

$$x = a(\cos\varphi_0)\theta + C_1, \qquad y = a(\sin\varphi_0)\theta + C_2, \tag{E.2}$$

the system (E.3) is no longer nonholonomic, it is holonomic. In such systems the relations between the generalized coordinates (e.g., (E.2)) are used to reduce the system to a lower-dimensional one free of constraints. Indeed, without loss of generality we can set $C_1 = C_2 = 0$ (this effectively defines the origin of the coordinate system used to locate the center of mass of the wheelbarrow) and substitute the resulting relations from (E.2) into the Lagrangian in (E.3). Simplifying the result yields

$$L_{\mathrm{h}} = \frac{1}{2}\beta\dot{\theta}^2. \tag{E.3}$$

The quantum mechanics of this system is identical to that of a quantum particle on a one-dimensional ring. The associated reduced energy spectrum is equation (33) in our article.

F Other Special Cases of Interest

F.1 The No Rolling Cases

Suppose we now remove the nonholonomic constraints in (1). The resulting system is defined by the Lagrangian $L - V$, where L is the Lagrangian in (1) and $V(\theta_{\mathrm{d}}) = \alpha[1 + \cos\theta_{\mathrm{d}}]$. Physically, this model describes the dynamics of a *sliding* wheelbarrow (if on a surface), or if not on a surface, a wheelbarrow whose body can rotate in the xy-plane but not in the z-direction. (Notably, relative wheel rotation does not lead to translational motion here, because the angles θ_{d} and φ are only related in the rolling case, via the last constraint in (1).) The equations of motion in this case are

$$I\ddot{\theta}_{\mathrm{d}} = 4\alpha\sin(\theta_{\mathrm{d}}), \qquad I\ddot{\theta} = 0, \qquad I_1\ddot{\varphi} = 0, \qquad m\ddot{x} = 0, \qquad m\ddot{y} = 0, \tag{F.1}$$

whose x- and y-solutions are linear in t. (Thus, this unconstrained system, unlike its nonholonomic counterpart, can never have circular trajectories in the xy-plane.) The corresponding Hamiltonian is:

$$H_{\mathrm{nr}} = \frac{1}{2}\left[\frac{p_\varphi^2}{I_1} + \frac{p_\theta^2}{I} + \frac{4p_{\theta_{\mathrm{d}}}^2}{I} + \frac{1}{m}\left(p_x^2 + p_y^2\right)\right] + \alpha[1 + \cos\theta_{\mathrm{d}}]. \tag{F.2}$$

The kinetic energy metric of L_{nr} is $g_{\mathrm{nr}} = diag(I_1, I, I/4, m, m)$ (relative to the coordinate ordering $(\varphi, \theta, \theta_{\mathrm{d}}, x, y)$) and is positive definite. A straightforward check of the other requirements for geometric quantization shows that they are satisfied. The Hamiltonian operator is once again given by (15), but in this case the R-correction is zero. A straightforward calculation yields an energy spectrum similar to (26) for the same $\mu_x = \mu_y = 0$ case:

$$\widetilde{E}^{\mathrm{nr}}_{(k,l,n)} = \alpha + \frac{\hbar^2 k^2}{2I_1} + \frac{\hbar^2 l^2}{2I} + \frac{\hbar^2}{2I}c_n(\gamma^{\mathrm{nr}}), \quad \gamma^{\mathrm{nr}} = \frac{I\alpha}{\hbar^2}, \quad k, l \in \mathbb{Z}, \quad n = 0, 1, \ldots, \quad \widetilde{E}^{\mathrm{nr}}_{(0,0,0)} \approx \hbar\sqrt{\frac{\alpha}{I}}, \tag{F.3}$$

where for the last approximation we assumed large γ and again used the approximations (27). In the zero-potential case (i.e., $\alpha = 0$), we obtain the energies

$$\widetilde{E}^{\mathrm{nr},0}_{(k,l,j)} = \frac{\hbar^2}{2}\left(\frac{k^2}{I_1} + \frac{l^2}{I} + \frac{4j^2}{I}\right), \quad k, l, j \in \mathbb{Z}, \quad \widetilde{E}^{\mathrm{nr},0}_{(0,0,0)} = 0. \tag{F.4}$$

(We have appended the superscript "nr,0" to distinguish this unconstrained, zero potential energy spectrum from the others.)

F.2 The Rolling, Nonholonomic Case with Zero Potential

Consider the wheelbarrow variant described in the penultimate paragraph of the Discussion section: a wheelbarrow subject to the nonholonomic constraints in (2) yet whose intramolecular rotation generates no potential (i.e., $\alpha = 0$). Such a system is a nonholonomic system modeled by (5) with $\alpha = 0$. Setting $\alpha = 0$ in (19) produces energies similar to those in (31), except that the R-correction from (17) is present:

$$\widetilde{E}^{\mathrm{nh},0}_{(k,l)} = \frac{\hbar^2}{2}\left(\frac{k^2}{J} + \frac{l^2}{\beta}\right) - \frac{ma^2\hbar^2}{24\beta J}, \quad k, l \in \mathbb{Z}, \quad \widetilde{E}^{\mathrm{nh},0}_{(0,0)} = -\frac{ma^2\hbar^2}{24\beta J}. \tag{F.5}$$

References

[1] Bloch, A.M. (2003). *Nonholonomic mechanics and control*. New York: Springer.

[2] Cortes, J. (2002). *Geometric, control and numerical aspects of nonholonomic systems*. New York: Springer.

[3] Grosso, G. and Parravicini, G.P. (2000). *Solid State Physics*. San Diego, CA: Academic Press.
